# Back Pain Prevalence and Its Associated Factors in Brazilian Athletes from Public High Schools: A Cross-Sectional Study

**DOI:** 10.1371/journal.pone.0150542

**Published:** 2016-03-03

**Authors:** Matias Noll, Ivan Silveira de Avelar, Georgia Cristina Lehnen, Marcus Fraga Vieira

**Affiliations:** 1 Bioengineering and Biomechanics Laboratory, Universidade Federal de Goiás, Goiânia, Goiás, Brazil; 2 Instituto Federal Goiano–Campus Ceres, Ceres, Goiás, Brazil; The James Cook University Hospital, UNITED KINGDOM

## Abstract

Most studies on the prevalence of back pain have evaluated it in developed countries (Human Development Index—HDI > 0.808), and their conclusions may not hold for developing countries. The aim of this study was to identify the prevalence of back pain in representative Brazilian athletes from public high schools. This cross-sectional study was performed during the state phase of the 2015 Jogos dos Institutos Federais (JIF), or Federal Institutes Games, in Brazil (HDI = 0.744), and it enrolled 251 athletes, 173 males and 78 females (14–20 years old). The dependent variable was back pain, and the independent variables were demographic, socioeconomic, psychosocial, hereditary, exercise-level, anthropometric, strength, behavioral, and postural factors. The prevalence ratio (PR) was calculated using multivariable analysis according to the Poisson regression model (α = 0.05). The prevalence of back pain in the three months prior to the study was 43.7% (n = 104), and 26% of the athletes reported feeling back pain only once. Multivariable analysis showed that back pain was associated with demographic (sex), psychosocial (loneliness and loss of sleep in the previous year), hereditary (ethnicity, parental back pain), strength (lumbar and hand forces), anthropometric (body mass index), behavioral (sleeping time per night, reading and studying in bed, smoking habits in the previous month), and postural (sitting posture while writing, while on a bench, and while using a computer) variables. Participants who recorded higher levels of lumbar and manual forces reported a lower prevalence of back pain (PR < 0.79), whereas feeling lonely in the previous year, obesity, and ethnicity exhibited the highest prevalence ratio (PR > 1.30). In conclusion, there is no association between exercise levels and back pain but there is an association between back pain and non-exercise related variables.

## Introduction

In global terms, back pain is a common complaint in industrialized societies, affecting between 54% and 90% of the adult population [1]. In Brazil, the 2014 National Health Survey, conducted by the Ministry of Health together with the Brazilian Institute of Geography and Statistics, identified 27 million adults affected by chronic spinal diseases, which corresponds to 18.5% of the adult population [2].

In addition to being widespread among adults [3–6], back pain is also reported in children and adolescents [7–9], frequently affecting more than 50% of Brazilian schoolchildren [10,11]. Although the relationship between the existence of back pain in adolescents and in adults is well established and the presence of this disorder in adolescence increases its chronicity in adulthood [12–14], in developing countries such as Brazil (Human Development Index—HDI = 0.744), this health problem has been neglected and more research on it is needed [15]. Prevalence studies give an indication of the extent of such health problems in a specific population, and they are useful as starting points for future epidemiological and intervention studies designed to prevent and reduce the impacts of this health problem [16].

Back pain in adolescents is widely understood as having multiple causes, including physical, behavioral, genetic, and psychosocial factors [17–19]. In addition, the increased prevalence of back pain in adolescents may be related to their increasing level of participation in organized sports [20,21]. To the best of our knowledge, the research on this issue in Brazil and South America has focused on training and nutritional factors [22–26] and sport injuries [27–29]; no studies have investigated the prevalence of back pain among Brazilian high school athletes or high school athletes in other South American countries. Because most studies of back pain prevalence have targeted developed countries (HDI > 0.808) [7,20,30], their conclusions may not hold for developing countries [16,31].

A continental country, Brazil consists of regions with varying characteristics, and many adolescent athletes do not live under optimal health and educational conditions [32]. A 2013 survey conducted in Brazil by the Sports Ministry revealed that 25% of the adolescent population (15–19 years old) played sports and that 83.7% of this population did not receive any instruction for practice or did not have a coach [33]. Approximately 12% of all players—equivalent to over 2.1 million athletes—participated in competitions annually [32]. Altogether, these data reveal the precarious training conditions to which Brazilian adolescents are subjected, such as lack of regular medical care, lack of proper nutrition, and inappropriate sporting equipment, a fact that reinforces the need for prevalence studies of this population [34].

These concerns are relevant especially in view of the upcoming 2016 Olympic Games in Rio de Janeiro, because athletic training is being strongly encouraged by the media and by federal and state governmental agencies, such as the Sports Ministry and the Brazilian Olympic Committee. Participation in high school sports has been promoted by several public scholarships and competitions [35,36]. These competitions are held annually and are a method of selecting the best athletes for the sport clubs and Brazilian national teams.

In addition to the factors commonly studied in the literature, such as type of sport, gender, and training intensity and frequency [30,37,38], it is necessary to evaluate the relationship between back pain and factors such as strength and the asymmetric distribution of biomechanical loads, which are fundamental aspects of sports performance and motor control [39]. We investigated both these factors and certain health variables related to demographic, socioeconomic, psychosocial, hereditary, behavioral, and postural factors.

The present study aims to contribute to a better understanding of back pain prevalence and its related factors in high school athletes, providing the insights necessary to preventing it and properly advising the athletes.

The objectives are:

1. To identify the prevalence of back pain in Brazilian athletes from public high schools;
2. To measure the prevalence of possible associated variables such as: demographic, socioeconomic, psychosocial, hereditary, exercise-level, anthropometric, strength, behavioral, and postural factors;
3. To perform multivariable statistical analysis on these variables.

## Methods

This cross-sectional study was performed during the state phase of the 2015 Jogos dos Institutos Federais (JIF), or the Federal Institutes Games, in Brazil. A total of 361 athletes, representing 14 cities of the state of Goiás, in the Brazilian Midwest, took part in the event. The athletes were high school students regularly enrolled in federal institutes in the state of Goiás, and they were a representative sample of public high school students that regularly practice sports. The JIF are organized annually, and the regional and national phases, for which the top-ranked athletes are selected, follow the state phase.

The inclusion criteria for this study were as follows: 14–20 years old, no previous history of musculoskeletal surgery, and participation in one of the following sport modalities: volleyball, basketball, handball, or soccer. A total of 320 athletes met the inclusion criteria and were invited to participate in this study. Of these athletes, 42 declined to participate and 27 were injured. Consequently, the study enrolled 251 athletes, 173 males and 78 females (16.4 ± 1.4 years old, 65.2 ± 12.1 kg, 1.71 ± 0.09 m). Table 1 presents the frequency and percentage of participants according to sex and age.

**Table 1:** Frequency and percentage of high school athletes categorized by sex and age.

Age	Male	Female	Total
	n (%)	n (%)	n (%)
14	7 (58.3)	5 (41.7)	12 (100)
15	31 (54.4)	26 (45.6)	57 (100)
16	53 (70.7)	22 (29.3)	75 (100)
17	44 (80)	11 (20)	55 (100)
18	22 (78.6)	6 (21.4)	28 (100)
19	11 (68.8)	5 (31.2)	16 (100)
20	5 (62.5)	3 (37.5)	8 (100)
Total	173 (68.9)	78 (31.1)	251 (100)

The present study is in accordance with the Helsinki Declaration and was approved by the Ethics Committee for Human Research of the Universidade Federal de Goiás. The athletes were allowed to leave the study at will and to opt out of any of our procedures. Prior to participation, the athletes, and their parents or guardians, in the case of minors, voluntarily signed an informed consent form approved specifically for this study.

### Questionnaires

To determine the prevalence of back pain and behavioral and postural habits, a self-administered questionnaire entitled “Back Pain and Body Posture Evaluation Instrument” (BackPEI) was used, which is a valid and reproducible questionnaire consisting of 21 closed questions with a different version for each sex [40].

The questionnaire addressed the following issues: (1) back pain in the previous three months (occurrence and frequency), (2) demographic factors (age and sex), (3) behavioral factors (reading/studying in bed; time spent watching television, using a computer, and sleeping per day), (4) postural factors (sitting posture when writing, using a computer, and talking; posture when using a backpack; and sleeping position), (5) hereditary factors (occurrence of back pain in parents), and (6) level of exercise practice (frequency, sport modality, competition).

The questions concerning sitting posture when writing, using a computer, and talking, as well as the postures adopted when lifting an object from the floor and carrying school materials, were illustrated with pictures of subjects performing those activities. For statistical analysis, only one alternative was considered correct, whereas the remaining alternatives were grouped as incorrect [40]. The reliability of the questionnaire was tested with 34 high school students who did not participate in the study. A test-retest protocol with seven days interval was used, and good and very good values [41] for all questions were verified (Kappa range: 0.704 to 0.944).

The Brazilian National School-Based Health Survey (PeNSE), a validated and reproducible questionnaire [42,43], was used to assess the following factors: socioeconomic (“Do you currently have a job?”; answers: “yes” or “no”), psychosocial (“How frequently did you feel lonely last year?”; “How frequently did you lose sleep last year due to something that concerned you?”; “How frequently did you feel intimidated last month?”; answers: “never”, “rarely”, “sometimes”, or “almost every day”), hereditary (“Choose your ethnicity”; answers: “White”, “Black”, “Asian”, “Mulatto”, or “Indigenous”), and behavioral (“Did you smoke frequently last month?”; “Did you consume alcohol frequently last month?”; answers: “yes” or “no”).

The questionnaire’s reliability was tested with 34 high school students who did not participate in the study, and again, good and very good values [41] for all questions were verified (Kappa range: 0.701 to 0.841).

At the outset of the study, the researcher explained to the subjects as a group how both questionnaires should be answered. The subjects then answered the questionnaires individually. The responses that the athletes gave were anonymous.

### Anthropometry

Each athlete’s body mass and height were measured. Body mass index (BMI) (kg/m^2^) was calculated by dividing the mass (kg) by the square height (m^2^). Athletes were classified according to standard deviation (SD) of BMI Z-score as normal weight (−2SD < BMI Z-score < 1SD), overweight (1SD ≤ BMI Z-score < 2SD), or obese (BMI Z-score ≥ 2SD), based on the World Health Organization’s Growth Reference [44].

### Manual and Lumbar Force

Manual and lumbar forces were evaluated using a manual force dynamometer (EMG SYSTEM, TRF_MAN model) and a lumbar force dynamometer (EMG SYSTEM, TRF_ELMB model), respectively (nominal capacity 200 kg, sensitivity 2 mV/V ± 10%, error < 0.03%, input resistance 405 Ω, output resistance 350 Ω). Both dynamometers were calibrated before data collection and were adjusted according to the size of each athlete. The data were collected using a signal conditioner with 16 channels and 100x amplification and an A/D converter with 16-bit resolution.

For the collection of manual force data, the athlete sat with an elbow flexion of 90°, shoulders adducted and neutrally rotated, forearm in neutral position, and wrist with a 10° extension [45,46]. The manual force asymmetry index (MFAI) was calculated as follows (Eq 1):

$$MFAI=\frac{\text{ (RMF-LMF)}}{\text{ (RMF+LMF)}}\;\times \text{ 100\%}$$ (1)

where MFAI is the asymmetry index for manual force, RMF is the right manual force, and LMF is the left manual force.

The athlete executed two valid trials of 5 s for right manual force, left manual force, and lumbar force, and the largest value was computed. A resting period of 60 s between each trial was allowed. Lumbar and manual forces were normalized by body weight (BW).

As with the questionnaires, the reliability of strength data was tested with 34 high school students who did not participate in the study, and a high intraclass correlation coefficient (ICC) for the manual force dynamometer (ICC = 0.974, 0.969 to 0.981) and lumbar force dynamometer (ICC = 0.952, 0.939 to 0.962) was verified.

### Weight Asymmetry

The weight distribution was evaluated with two force plates (Dual-Top AccuSway AMTI model; 6 channels digital output, capacity 1112 N/136 Nm, sensitivity 0.67 μV/VN, natural frequency 120 Hz). It was assessed while the athlete stood for 30 s with one foot on each force plate, looking at a fixed point. The first 10 s were discarded, and the average weight under each foot was calculated for the remaining 20 s. The weight asymmetry index (WAI) was calculated using a custom-written MatLab code as shown in Eq 2:

$$WAI=\;\frac{\text{(RFW-LFW)}}{\text{(RFW+LFW)}}\;\times \text{ 100\%}$$ (2)

where WAI is the weight asymmetry index, RFW is the average weight under the right foot, and LFW is the average weight under the left foot.

### Statistical Analysis

The athletes were grouped according to their age as follows: 14 years to 15 years and 11 months; 16 years to 17 years and 11 months; and 18 to 20 years. The data for lumbar force, the MFAI, and the WAI were divided into four groups according to quartiles.

Data were analyzed using descriptive statistics and the chi-squared test of association (bivariate analysis) for the dependent variable “back pain”; the demographic, socioeconomic, psychosocial, hereditary, exercise-level, anthropometric, strength, behavioral, and postural factors were considered independent variables. For all analyses, sex and age were considered covariates. The independent variables with a level of significance of *p* < 0.20 in the bivariate analysis were included in a Poisson regression model with robust variance. The measure of effect was the prevalence ratio with its respective 95% confidence intervals (CIs). All statistical analyses were performed using the Statistical Package for the Social Sciences version 20.0, with the level of significance set at α = 0.05.

## Results

The prevalence of back pain in the three months prior to the study was 43.7% (n = 104). Thirteen athletes (5.2%) reported not know clearly if they had or not back pain (they chose the alternative “I don’t know”), and 134 (51.1%) did not report back pain. The thirteen athletes (n = 8, corresponding 4.6% from all male athletes; n = 4, corresponding 5.1% from all female athletes) were excluded from the sample not affecting the results (they presented similar descriptive results that all included athletes).

Table 2 presents the descriptive data for the athletes’ back pain frequency and limitations in daily activities.

**Table 2:** Back pain frequency in the three months prior to the study and limitations when performing daily activities (n = 104, 43.7% of total sample).

Variable	Male	Female	Total
	n (%)	n (%)	n (%)
**Frequency**			
Only once	18 (30)	9 (20.5)	27 (26)
Once a month	11 (18.3)	6 (13.6)	17 (16.3)
Once a week	9 (15)	9 (20.5)	18 (17.3)
Twice to three times per week	5 (8.3)	6 (13.6)	11 (10.6)
Four or more times per week	0 (0)	6 (13.6)	6 (5.8)
No answer	17 (28.4)	8 (18.2)	25 (24)
**Limitations when performing daily activities**			
Yes	2 (3.3)	7 (15.9)	9 (8.7)
No	54 (90)	36 (81.8)	90 (86.5)
No answer	4 (6.7)	1 (2.3)	5 (4.8)

In total 33.7% of athletes experienced back pain one or more times per week. Most athletes reported feeling back pain only once in the previous three months and once a month (42.3%), with females reporting a higher frequency of back pain than males. Back pain imposed limitations when performing daily activities in 8.7% of the athletes, and this variable exhibited greater frequency in female (15.9%) than in male athletes (3.3%).

Descriptive results from manual force, lumbar force, the MFAI, and the WAI are presented in Table 3.

**Table 3:** Descriptive results from lumbar force, manual force, MFAI, and WAI.

Independent variable	Male	Female
	Mean ± SD	Mean ± SD
*Lumbar force normalized by BW*		
14–15 years	1.78 ± 0.38	1.17 ± 0.28
16–17 years	1.63 ± 0.33	1.16 ± 0.22
18–20 years	1.70 ± 0.31	1.16 ± 0.27
*Manual force normalized by BW—right hand*		
14–15 years	0.63 ± 0.13	0.44 ± 0.92
16–17 years	0.64 ± 0.12	0.42 ± 0.11
18–20 years	0.64 ± 0.13	0.43 ± 0.11
*Manual force asymmetry index (%)*		
14–15 years	5.21 ± 6.14	7.23 ± 4.98
16–17 years	5.56 ± 3.65	6.58 ± 4.41
18–20 years	5.96 ± 4.68	7.72 ± 3.97
*Weight asymmetry index (%)*		
14–15 years	6.28 ± 5.70	5.91 ± 3.74
16–17 years	6.85 ± 6.38	5.66 ± 4.38
18–20 years	7.67 ± 6.59	5.92 ± 8.25

Multivariable analysis revealed a significant association between back pain and demographic (sex), psychosocial (feeling lonely, loss of sleep due to a concerning issue), hereditary (ethnicity, parents with back pain), anthropometric (BMI), strength (manual and lumbar force), behavioral (time sleeping per night, reading/studying in bed, smoking habits) and postural (sitting posture when writing, using a computer, and talking) variables (Tables 4–7).

**Table 4:** Association (χ^2^) and prevalence ratio between back pain and independent variables (demographic, socioeconomic, and psychosocial).

Independent variable	n (%)	Back pain	Prevalence ratio	*p* ^a^
		n (%)	(95% CI)	
**Demographic**				
*Sex (n = 238)*				*0*.*001*^b^
Male	165 (69.3)	60 (36.4)	1	
Female	73 (30.7)	44 (60.3)	1.17 (1.07–1.28)	
*Age (n = 238)*				*0*.*167*
14–15 years	66 (27.7)	24 (36.4)	1	
16–17 years	121 (50.9)	60 (49.6)	1.09 (0.98–1.21)	
18–20 years	51 (21.4)	20 (39.2)	1.02 (0.89–1.16)	
**Socioeconomic**				
*Work (n = 235)*				*0*.*912*
Yes	28 (11.9)	12 (42.9)	1	
No	207 (88.1)	91 (44)	1.01 (0.87–1.15)	
*Mother education (n = 221)*				*0*.*964*
Basic education	52 (23.5)	24 (46.2)	1	
High school	95 (40)	42 (44.2)	0.98 (0.87–1.10)	
College	74 (33.5)	34 (45.9)	0.99 (0.88–1.12)	
*Father education (n = 206)*				*0*.*696*
Basic education	92 (44.7)	45 (48.9)	1	
High school	86 (41.7)	37 (43)	0.96 (0.86–1.06)	
College	28 (13.6)	12 (42.9)	0.95 (0.82–1.11)	
**Psychosocial**				
*Feeling lonely last year (n = 224)*				*0*.*002*^b^
Never	69 (30.8)	25 (36.2)	1	
Rarely	85 (38)	36 (42.4)	1.04 (0.93–1.16)	
Sometimes	59 (26.3)	30 (50.8)	1.11 (0.98–1.16)	
Almost every day	11 (4.9)	9 (81.8)	1.33 (1.14–1.55)	
*Loss of sleep last year (n = 223)*				*0*.*002*^b^
Never	80 (35.9)	24 (30)	1	
Rarely	84 (37.7)	39 (46.4)	1.12 (1.01–1.25)	
Sometimes	46 (20.6)	28 (60.9)	1.23 (1.10–1.39)	
Almost every day	13 (5.8)	8 (61.5)	1.24 (1.03–1.48)	
*Feeling intimidated last month (n = 228)*				*0*.*322*
Never	130 (57)	53 (40.8)	1	
Rarely	57 (25)	26 (45.6)	1.03 (0.92–1.15)	
Sometimes	21 (9.2)	11 (52.4)	1.08 (0.92–1.26)	
Almost every day	20 (8.8)	12 (60)	1.13 (0.98–1.31)	

^a^ Multivariable analysis. Wald chi-squared test.

^b^ Significant association (*p* < 0.05).

**Table 5:** Association (χ^2^) and prevalence ratio between back pain and independent variables (hereditary, anthropometry, and strength).

Independent variable	n (%)	Back pain	Prevalence ratio	*p* ^a^
		n (%)	(95% CI)	
**Hereditary**				
*Ethnicity (n = 235)*				*> 0*.*001*^b^
White	72 (30.7)	29 (40.3)	1	
Black	36 (15.3)	15 (41.7)	1.01 (0.87–1.16)	
Asian	16 (6.8)	11 (68.8)	1.20 (1.02–1.41)	
Mulatto	106 (45.1)	43 (40.6)	1.01 (0.90–1.11)	
Indigenous	5 (2.1)	5 (100)	1.42 (1.31–1.54)	
*Parents with back pain (n = 181)*				*0*.*002*^b^
No	69 (38.1)	20 (29)	1	
Yes	112 (61.9)	58 (51.8)	1.17 (1.06–1.31)	
**Anthropometry**				
*Body mass index (n = 235)*				*0*.*002*^b^
Normal weight	172 (73.2)	70 (40.7)	1	
Overweight	56 (23.8)	28 (50)	1.06 (0.96–1.18)	
Obese	7 (3)	6 (85.7)	1.32 (1.14–1.43)	
*Weight asymmetry index (n = 198)*				*0*.*754*
1^st^ Quartile—lowest	47 (23.7)	20 (42.6)	1	
2^nd^ Quartile	49 (24.7)	23 (46.9)	1.03 (0.89–1.18)	
3^rd^ Quartile	51 (25.8)	25 (49)	1.04 (0.91–1.19)	
4^th^ Quartile—highest	51 (25.8)	20 (39.2)	0.97 (0.85–1.12)	
**Strength**				
*Lumbar force normalized by BW (n = 235)*				*>0*.*001*^b^
1^st^ Quartile—lowest	58 (24.7)	39 (67.2)	1	
2^nd^ Quartile	61 (26)	25 (41)	0.84 (0.75–0.94)	
3^rd^ Quartile	56 (23.8)	22 (39)	0.83 (0.74–0.93)	
4^th^ Quartile—highest	60 (25.5)	18 (30)	0.77 (0.69–0.87)	
*Manual force of right hand normalized by BW (n = 235)*				*>0*.*001*^b^
1^st^ Quartile—lowest	60 (25.5)	39 (65)	1	
2^nd^ Quartile	56 (23.9)	26 (46.4)	0.88 (0.79–0.99)	
3^rd^ Quartile	60 (25.5)	22 (36.7)	0.82 (0.74–0.93)	
4^th^ Quartile—highest	59 (25.1)	17 (28.8)	0.78 (0.69–0.87)	
*Manual force asymmetry index (n = 235)*				*0*.*895*
1^st^ Quartile—lowest	61 (26)	26 (42.6)	1	
2^nd^ Quartile	56 (23.8)	23 (41.1)	0.99 (0.87–1.12)	
3^rd^ Quartile	61 (26)	29 (47.5)	1.03 (0.91–1.16)	
4^th^ Quartile—highest	57 (24.2)	26 (45.6)	1.02 (0.91–1.15)	

^a^ Multivariable analysis. Wald chi-squared test.

^b^ Significant association (*p* < 0.05).

**Table 6:** Association (χ^2^) and prevalence ratio between back pain and independent variable (exercise level and behavioral).

Independent variable	n (%)	Back pain	Prevalence ratio	*p* ^a^
		n (%)	(95% CI)	
**Exercise level**				
*Physical exercise weekly frequency (n = 215)*				0.888
1–2 days	75 (34.9)	33 (44)	1	
3 or more days	140 (65.1)	63 (45)	1.01 (0.91–1.11)	
*Sport modality (n = 217)*				*0*.*234*
Handball	34 (15.7)	19 (55.9)	1	
Volleyball	57 (26.2)	27 (47.4)	0.94 (0.82–1.08)	
Soccer	95 (43.8)	37 (38.9)	0.89 (0.78–1.01)	
Basketball	31 (14.3)	11 (35.5)	0.86 (0.73–1.02)	
*Competition (n = 227)*				*0*.*142*
Only 1 per year	109 (48)	53 (48.6)	1	
2 or more per year	118 (52)	46 (39)	0.93 (0.85–1.02)	
**Behavioral**				
*Time spent watching television per day (n = 219)*				*0*.*886*
0–1 hours	138 (63)	59 (42.8)	1	
2–3 hours	69 (31.5)	30 (43.5)	1.01 (0.91–1.11)	
4 or more hours	12 (5.5)	6 (50)	1.05 (0.86–1.28)	
*Time spent using computer per day (n = 204)*				*0*.*56*
0–1 hours	99 (48.5)	36 (36.4)	1	
2 or more hours	105 (51.5)	52 (49.5)	1.09 (0.99–1.21)	
*Time sleeping per night (n = 236)*				*0*.*042*^b^
0–7 hours	160 (67.8)	78 (48.8)	1	
8–9 hours (adequate)	51 (21.6)	15 (29.4)	0.87 (0.78–0.97)	
10 or more hours	25 (10.6)	10 (40)	0.94 (0.81–1.09)	
*Reading/studying in bed (n = 238)*				*0*.*042*^b^
Yes	94 (39.5)	49 (52.1)	1	
Sometimes	90 (37.8)	38 (42.2)	0.93 (0.84–1.03)	
No	54 (22.7)	17 (31.5)	0.86 (0.77–0.97)	
*Smoking habits last month (n = 238)*				*0*.*028*^b^
No	225 (94.5)	95 (42.2)	1	
Yes	13 (5.5)	9 (69.2)	1.19 (1.01–1.38)	
*Alcohol consumption last month (n = 238)*				*0*.*101*
No	162 (68.1)	65 (40.1)	1	
Yes	76 (31.9)	39 (51.3)	1.08 (0.98–1.18)	

^a^ Multivariable analysis. Wald chi-squared test.

^b^ Significant association (*p* < 0.05).

**Table 7:** Association (χ^2^) and prevalence ratio between back pain and independent variable (postural).

Independent variable	n (%)	Back pain	Prevalence ratio	*p* ^a^
		n (%)	(95% CI)	
**Postural**				
*Sleeping posture (n = 211)*				*0*.*481*
Supine	22 (10.4)	7 (31.8)	1	
Lateral decubitus	85 (40.3)	39 (45.9)	1.11 (0.94–1.31)	
Prone	104 (49.3)	44 (42.3)	1.08 (0.91–1.26)	
*Sitting posture to write (n = 238)*				*0*.*017*^b^
Recommended	16 (6.7)	3 (18.8)	1	
Not recommended	222 (93.3)	101 (45.5)	1.22 (1.03–1.44)	
*Sitting posture on a bench (n = 238)*				*0*.*001*^b^
Recommended	21 (8.8)	3 (14.3)	1	
Not recommended	217 (91.2)	101 (46.5)	1.28 (1.11–1.47)	
*Sitting posture to use computer (n = 238)*				*0*.*001*^b^
Recommended	40 (16.8)	8 (20)	1	
Not recommended	198 (83.2)	96 (48.5)	1.23 (1.10–1.38)	
*Posture adopted to lift object from floor (n = 238)*				*0*.*221*
Recommended	40 (16.8)	14 (35)	1	
Not recommended	198 (83.2)	90 (45.5)	1.07 (0.95–1.21)	
*Means of carrying school supplies (n = 238)*				*0*.*362*
Backpack	235 (98.7)	102 (43.4)	1	
Another (briefcase, purse, or other)	3 (1.3)	2 (66.7)	1.16 (0.84–1.61)	
*Method of carrying backpack (n = 238)*				*0*.*488*
Recommended (both shoulders)	168 (70.6)	71 (42.3)	1	
Not recommended	70 (29.4)	33 (47.1)	1.03 (0.94–1.13)	

^a^ Multivariable analysis. Wald chi-squared test.

^b^ Significant association (*p* < 0.05).

## Discussion

In this cross-sectional study, we focused on identifying back pain prevalence in high school athletes from Brazilian public schools.

The back pain prevalence in the three months prior to the study (43.7%) found in the studied population was similar to that found in studies of nonathlete students in Brazil (40%–60%). Noll et al. [11] evaluated 833 students from the state of Rio Grande do Sul using the BackPEI questionnaire, and they found a back pain prevalence of 54.1% in the previous three months. Two other recent studies, which evaluated back pain prevalence in Brazilian high school students from the states of Rio de Janeiro [10] and Rio Grande do Sul [47], found a back pain prevalence of 46.8% and 57%, respectively.

These rates of back pain prevalence are comparable to those found in developed countries for both athletes and nonathletes. Schmidt et al. [30], in a study of 272 competitive athletes from Germany aged 12 to 20 years, found a back pain prevalence of 57%. In Skoffer’s [48] study of the occurrence of pain in 546 students of both genders between 14 and 17 years of age in a town in Denmark, 51.3% of the students reported having felt pain in the three months prior to the study. Wirth et al. [7] evaluated 412 adolescents from Switzerland (10–16 years old) using a questionnaire and physical examination and found a back pain prevalence of 44.4%.

Furthermore, our results indicated that female athletes (60.3%) presented a higher prevalence of back pain than male athletes (36.4%). Many studies corroborate this finding, suggesting that female athletes have a higher predisposition for reporting back pain [18,20,49,50]. A possible explanation for this result may be the earlier maturity of girls (including hormonal changes during puberty) and their anatomical and functional characteristics (shorter stature, lower muscle and bone density) when compared to boys [50]. Moreover, due to social and educational factors, it may be more socially acceptable for women to reveal their symptoms and feelings than it is for men. Finally, as Shan et al. [18] reported, boys have a higher pain threshold than girls.

Similar results have been found in adults. Triki et al. [16] assessed back pain prevalence among student athletes (18–24 years old) who engaged in various sport modalities in Tunisia and found a significantly higher prevalence in female athletes than in male athletes. Legaults’ [37] results also indicate that female athletes presented a higher percentage of back injuries.

In the present study, approximately one-third of the athletes experienced back pain one or more times per week, and this was more common among females (47.7%) than males (23.3%). Furthermore, 8.7% of these athletes reported that back pain prevented them from performing daily activities, and this limitation affected more female athletes. The same arguments discussed in the previous paragraphs may be able to explain the differences among male and female athletes. On the other hand, Wirth et al. [7] reported that in the majority of subjects investigated in their study, back pain did not result in any consequences. However, similar to the present study, 55 subjects (30.1%) stated that they changed something in their daily lives due to their back pain, including taking pills, reducing their daily activities, or seeing a doctor.

A total of 26.8% of the athletes that participated in the present study were overweight or obese, and a significant association between obese and back pain was found. This level of prevalence is similar to that seen in other countries (~25%) with a similar population [51]. These weight characteristics may have a significant impact on the quality of life of these young people and may contribute to ongoing health problems, including musculoskeletal pain and bone/joint dysfunction in later life, and they may negatively influence aspects of motor performance, such as muscle strength, balance, and walking [51,52].

No association was found between back pain and age or between back pain and socioeconomic variables (work, parental education). In Brazil, a developing country, many adolescents work in addition to attending school [53]. Our results showed that 11% of the athletes had worked a job, but no association between this variable and back pain was found. However, we did not investigate specific types of work or the weekly frequency with which students had worked. Therefore, the influence of work on back pain and performance needs further study.

On the other hand, we found an association between back pain and psychosocial variables, which can interfere with athletes’ quality of life, causing depressive symptoms and stress that can in turn alter athletic performance [18]. Similarly, in a study with 1,446 students aged 11–14, Watson et al. [54] found that low back pain was strongly associated with emotional problems, behavioral problems, and general somatic complaints.

Significant biological, emotional, intellectual, and social changes take place during adolescence, and mental health problems are common in this age group [55]. There is an association between stressful life events and athletic injury, and psychosocial variables are strongly related to the development of pain and disability [56]. The psychosocial factors associated with back pain and disability include anxiety, distress, depression, and self-perceived poor health [39,56,57]. Similarly, Wirth et al. [7] showed that sleep disorders are one of the predictors of pain in multiple spinal areas. Sleep disorders in childhood that occur along with certain psychiatric disorders can lead to anxiety/depression disorders later in life [7].

Another finding is the association between back pain and hereditary variables (ethnicity and parents with back pain). In our study, athletes whose parents presented back pain had a higher probability of presenting back pain when compared to other athletes. We speculated that this association is due not only to genetic factors, but also to behavioral and/or psychosocial factors [14,58]. We found that Asian and Indigenous athletes presented higher back pain prevalence than other ethnic groups. However, because this association has been insufficiently investigated and our sample size is relatively small, further studies are necessary to confirm it.

We did not find an association between back pain and exercise (weekly frequency, sport modality, and competition). This result was unexpected, because several studies [16,29,39] have demonstrated an association between certain sport modalities and increased rates of back pain as well as a positive association between time spent practicing such modalities and increased rates of back pain. Sato el al. [20] conducted a cross-sectional study using a questionnaire to determine the level of back pain in 43,630 children and adolescents in Japan. They showed that the level of back pain was higher in athletes who spent a longer time participating in sport activities, suggesting that sport activity is a possible back pain factor. Sato et al. [20] also identified a significantly higher back pain prevalence for most popular sports, especially volleyball, judo, gymnastics, golf, and rugby when compared to wrestling.

Similarly, Triki et al. [16] showed that the sport modalities associated with higher rates of back pain prevalence were gymnastics, judo, handball, volleyball, and basketball. They hypothesized that this was as a result of the fatigue caused by the long periods of time spent practicing such activities. A recent study conducted by Noormohammadpour et al. [31] hypothesized that certain movements characteristic of these sport modalities, such as sudden and repetitive lumber flexion, hyperextension, rotation, and axial load, are associated with a higher incidence of pain among athletes. However, our results did not indicate any difference between handball, volleyball, soccer, and basketball athletes in terms of back pain prevalence. These contradictory results may be due to different levels of practice and training intensity between our sample and the cited study’s sample and/or to the different methods of investigation used in these studies.

Although the ideas that any subject could accurately identify their own posture and that there is one correct posture for everyone is controversial and contested in the current literature [59,60], we found an association between back pain and postural variables (sitting posture when writing, using a computer, and sitting on a bench). Students who usually sit with not recommended posture (forward trunk flexion, lack of lumbar support, and lack of forearm support) may be predisposed to higher levels of general discomfort [10,61–63]. Possible explanations for this situation may be the increase in pressure on intervertebral discs [64], which can (1) lead to disc malnutrition, (2) contribute to the development of general discomfort, such as pain, fatigue, and a spinal degenerative process, and (3) initiate mechanisms that can endanger the integrity of the musculoskeletal system, such as an imbalance between the passive, active, and neural systems responsible for the stability of the lumbopelvic region [9,62]. Possibly, these factors may contribute to the significant association found in the present study between back pain and not recommended sitting posture.

An association between these sitting postures and back pain was found in a similar study [11]. When reading or studying in bed or when writing or using a computer with not recommended posture (lordosed or kyphosed, overly arched or slouched), athletes may become vulnerable to increased intradiscal pressure [62,64] which, as previously discussed could contribute to back pain.

Our results indicate that athletes with higher lumbar force had lower back pain prevalence. Although our data are insufficient to support the hypothesis that this variable could be a protective factor, some studies suggest it. Sullivan et al. [65] showed that the activation of the superficial lumbar multifidus, internal oblique, and thoracic erector spinal muscles is important for trunk postural stabilization. Decreasing trunk muscle efficiency has been shown to increase the load on the lumbar discs and ligaments, leaving the lumbopelvic region vulnerable to strain, instability, or injury [65]. In other words, stronger lumbar muscles may decrease the chance of developing back pain.

An association between smoking and back pain was found. Similarly, in a recent meta-analysis, Shiri et al. [66] reported that current smokers have a higher prevalence and incidence of pain than nonsmokers. The authors also showed that this association is stronger in adolescents than in adults because the former may be more vulnerable to the adverse effects of smoking. A possible explanation for this association is that smoking may lead to reduced perfusion and malnutrition of the intervertebral discs and may increase the level of circulating proinflammatory cytokines, which signal the central nervous system and thereby lead to an amplification of pain [66,67].

## Limitations of the Study and Future Directions

Our findings should be interpreted with caution. Assessment by self-reported questionnaires has certain limitations, such as response bias caused by acquiescence, socially desirable responding, or extreme responding [37]. These types of response bias can lead to either overestimation or underestimation of a problem. Also it should be highlighted that BackPEI questionnaire as yet lacks thorough validity testing specifically with Brazilian athletes.

Although the use of the questionnaire with posture illustrations would be simpler in epidemiological research as well as in clinical applications, the lack of self-consciousness especially in adolescence can limit the assessment of posture habits using such questionnaires. Also the possibility of reverse causality needs to be raised—i.e. perhaps having back pain makes people more likely to believe their posture is poorer rather than people with back pain having poorer posture. Thus, the results should be interpreted carefully [10,40].

Furthermore, it is important to make clear that because this is a cross-sectional study, it does not provide information about the nature of the development of pain and its associated factors. In other words, no inferences about cause and effect can be made. Therefore, a longitudinal exploration of these variables would be of interest [55,68]. Regarding the associated factors, it is important to identify them and to discuss the possibility that they are potential risk or protective factors. Thus, future research should investigate these issues.

In conclusion, the prevalence of back pain in high school athletes in the three months prior to the study was 43.7%—and this is in keeping with rates in developed countries and in non-athletic populations. Furthermore, there is no association between exercise levels and back pain but there is an association between back pain and non-exercise related variables and these warrant further investigation.

## References

[1] Hoy D, Brooks P, Blyth F, Buchbinder R. The Epidemiology of low back pain. Best Practice and Research: Clinical Rheumatology. 2010 pp. 769–781. 10.1016/j.berh.2010.10.002 21665125

[2] Perceived health status, lifestyles and chronic diseases Brazil, Major Regions and Federation Units. National Survey of Health 2014.

[3] Quemelo PRV, Gasparato FDS, Vieira ER. Prevalence, risks and severity of musculoskeletal disorder symptoms among administrative employees of a Brazilian company. Work. IOS Press; 2015; Preprint: 1–8. 10.3233/WOR-152131 26409374

[4] Meucci RD, Fassa AG, Faria NMX, Fiori NS. Chronic low back pain among tobacco farmers in southern Brazil. Int J Occup Environ Health. 2015;21: 66–73. 10.1179/2049396714Y.0000000094 25633930 PMC4273522

[5] Alperovitch-Najenson D, Santo Y, Masharawi Y, Katz-Leurer M, Ushvaev D, Kalichman L. Low back pain among professional bus drivers: ergonomic and occupational-psychosocial risk factors. Isr Med Assoc J. 2010;12: 26–31. 20450125

[6] de Ceballos AGDC, Santos GB. Factors associated with musculoskeletal pain among teachers: sociodemographics aspects, general health and well-being at work. Rev Bras Epidemiol. 2015;18: 702–715. 10.1590/1980-5497201500030015 26247193

[7] Wirth B, Humphreys BK. Pain characteristics of adolescent spinal pain. BMC Pediatr. 2015;15: 42 10.1186/s12887-015-0344-5 25886130 PMC4411747

[8] Yao W, Mai X, Luo C, Ai F, Chen Q. A Cross-Sectional Survey of Nonspecific Low Back Pain Among 2083 Schoolchildren in China. Spine (Phila Pa 1976). 2011;36: 1885–1890. 21270687 10.1097/BRS.0b013e3181faadea

[9] Paananen MV., Auvinen JP, Taimela SP, Tammelin TH, Kantomaa MT, Ebeling HE, et al Psychosocial, mechanical, and metabolic factors in adolescents’ musculoskeletal pain in multiple locations: A cross-sectional study. Eur J Pain. 2010;14: 395–401. 10.1016/j.ejpain.2009.06.003 19640750

[10] Meziat Filho N, Coutinho ES, Azevedo e Silva G. Association between home posture habits and low back pain in high school adolescents. Eur Spine J. 2014;24: 425–433. 10.1007/s00586-014-3571-9 25212451

[11] Noll M, Candotti CT, Nichele B, Cristina M, Schoenell W, Tiggemann CL, et al Back pain and the postural and behavioral habits of students in the municipal school network of Teutônia, Rio Grande do Sul. J Hum Growth Dev. 2013;23: 129–135.

[12] Trigueiro MJ, Massada L, Garganta R. Back pain in Portuguese schoolchildren: Prevalence and risk factors. Eur J Public Health. 2013;23: 499–503. 10.1093/eurpub/cks105 22874731

[13] Trevelyan FC, Legg SJ. Back pain in school children—where to from here? Appl Ergon. 2006;37: 45–54. 10.1016/j.apergo.2004.02.008 16137636

[14] Balagué F, Mannion AF, Pellisé F, Cedraschi C. Non-specific low back pain. Lancet. 2012;379: 482–491. 10.1016/S0140-6736(11)60610-7 21982256

[15] Noll M, Vieira A, Darski C, Candotti CT. Escolas posturais desenvolvidas no Brasil: Revisão sobre os instrumentos de avaliação, as metodologias de intervenção e seus resultados. Rev Bras Reumatol. 2014;54: 51–58. 10.1016/j.rbr.2013.04.007 24878792

[16] Triki M, Koubaa A, Masmoudi L, Fellmann N, Tabka Z. Prevalence and risk factors of low back pain among undergraduate students of a sports and physical education institute in Tunisia. Libyan J Med. 2015;10: 26802 10.3402/ljm.v10.26802 25758252 PMC4355506

[17] Burton AK. How to prevent low back pain. Best Pract Res Clin Rheumatol. 2005;19: 541–555. 10.1016/j.berh.2005.03.001 15949775

[18] Shan Z, Deng G, Li J, Li Y, Zhang Y, Zhao Q. Correlational Analysis of neck/shoulder Pain and Low Back Pain with the Use of Digital Products, Physical Activity and Psychological Status among Adolescents in Shanghai. PLoS One. 2013;8 10.1371/journal.pone.0078109 PMC3795657 24147114

[19] Wedderkopp N, Kjaer P, Hestbaek L, Korsholm L. High-level physical activity in childhood seems to protect against low back pain in early adolescence. Spine J. Elsevier Inc.; 2009;9: 134–141. 10.1016/j.spinee.2008.02.003 18495545

[20] Sato T, Ito T, Hirano T, Morita O, Kikuchi R, Endo N, et al Low back pain in childhood and adolescence: Assessment of sports activities. Eur Spine J. 2011;20: 94–99. 10.1007/s00586-010-1485-8 20582557 PMC3036027

[21] Haus BM, Micheli LJ. Back Pain in the Pediatric and Adolescent Athlete. Clin Sports Med. Elsevier; 2012;31: 423–440. 10.1016/j.csm.2012.03.011 22657993

[22] Fortes L de S, Kakeshita IS, Almeida SS, Gomes AR, Ferreira MEC. Eating behaviours in youths: A comparison between female and male athletes and non-athletes. Scand J Med Sci Sports. 2014;24: e62–8. 10.1111/sms.12098 23889336

[23] Juzwiak CR, Amancio OMS, Vitalle MSS, Szejnfeld VL, Pinheiro MM. Effect of calcium intake, tennis playing, and body composition on bone-mineral density of Brazilian male adolescents. Int J Sport Nutr Exerc Metab. 2008;18: 524–38. http://www.ncbi.nlm.nih.gov/pubmed/19033613. 19033613 10.1123/ijsnem.18.5.524

[24] Gomes RV, Cunha VCR, Zourdos MC, Aoki MS, Moreira A, Fernandez-Fernandez J, et al Physiological responses of young tennis players to training drills and simulated match play. J Strength Cond Res. 2015; 10.1519/JSC.0000000000001159 26382129

[25] Silva RT, Takahashi R, Berra B, Cohen M, Matsumoto MH. Medical assistance at the Brazilian juniors tennis circuit—a one-year prospective study. J Sci Med Sport. 2003;6: 14–8. 10.1016/S1440-2440(03)80004-X 12801206

[26] Silva CC, Goldberg TBL, Teixeira AS, Dalmas JC. The impact of different types of physical activity on total and regional bone mineral density in young Brazilian athletes. J Sports Sci. 2011;29: 227–34. 10.1080/02640414.2010.529456 21170799

[27] Almeida GPL, de Souza VL, Sano SS, Saccol MF, Cohen M. Comparison of hip rotation range of motion in judo athletes with and without history of low back pain. Man Ther. 2012;17: 231–5. 10.1016/j.math.2012.01.004 22281524

[28] Bastos FDN. Sports Injuries among Young Basketball Players: A Retrospective Study. J Clin Trials. 2014;04 10.4172/2167-0870.1000173

[29] Vanderlei F, Bastos F, Tsutsumi GY, Vanderlei LC, Netto J, Pastre C. Characteristics and contributing factors related to sports injuries in young volleyball players. BMC Res Notes. 2013;6: 415 10.1186/1756-0500-6-415 24124803 PMC4015734

[30] Schmidt CP, Zwingenberger S, Walther A, Reuter U, Kasten P, Seifert J, et al Prevalence of low back pain in adolescent athletes—An epidemiological investigation. Int J Sports Med. 2014;35: 684–689. 10.1055/s-0033-1358731 24424960

[31] Noormohammadpour P, Rostami M, Mansournia MA, Farahbakhsh F, Pourgharib Shahi MH, Kordi R. Low back pain status of female university students in relation to different sport activities. Eur Spine J. 2015; 10.1007/s00586-015-4034-7 26026471

[32] IBGE :: Instituto Brasileiro de Geografia e Estatística [Internet]. [cited 11 Nov 2015]. Available: http://www.ibge.gov.br/home/. Accessed 11 November 2015.

[33] Diagnóstico Nacional do Esporte [Internet]. [cited 11 Nov 2015]. Available: http://www.esporte.gov.br/diesporte/2.html. Accessed 11 November 2015.

[34] De Luigi AJ. Low Back Pain in the Adolescent Athlete. Phys Med Rehabil Clin N Am. Elsevier Inc; 2014;25: 763–788. 10.1016/j.pmr.2014.06.004 25442158

[35] Ministério do Esporte [Internet]. [cited 11 Nov 2015]. Available: http://www.esporte.gov.br/. Accessed 11 November 2015.

[36] Comitê Olímpico do Brasil—COB [Internet]. [cited 11 Nov 2015]. Available: http://www.cob.org.br/en. Accessed 11 November 2015.

[37] Legault ÉP, Descarreaux M, Cantin V. Musculoskeletal symptoms in an adolescent athlete population: a comparative study. BMC Musculoskelet Disord. BMC Musculoskeletal Disorders; 2015;16: 210 10.1186/s12891-015-0681-4 26285701 PMC4543469

[38] Mortazavi J, Zebardast J, Mirzashahi B. Low Back Pain in Athletes. Asian J Sports Med. 2015;6: 1–8. 10.5812/asjsm.6(2)2015.24718 PMC4592766 26448841

[39] Bizzini M, Junge A, Dvorak J. Sports Injuries and Prevention. Sport Inj Prev. 2015; 199–208. 10.1007/978-4-431-55318-2

[40] Noll M, Tarragô Candotti C, Vieira A, Fagundes Loss J. Back Pain and Body Posture Evaluation Instrument (BackPEI): Development, content validation and reproducibility. Int J Public Health. 2013;58: 565–572. 10.1007/s00038-012-0434-1 23275945

[41] Altmann DG. Practical statistics for medical research. Boca Raton Chapman and Hall/CRC; 1999.

[42] Tavares LF, de Castro IRR, Levy RB, Cardoso L de O, Claro RM. Dietary patterns of Brazilian adolescents: results of the Brazilian National School-Based Health Survey (PeNSE). Cad Saude Publica. Escola Nacional de Saúde Pública, Fundação Oswaldo Cruz; 2014;30: 2679–90. 10.1590/0102-311X00016814 26247996

[43] Brasil. Ibge. Pesquisa Nacional de Saúde—2013: percepção do estado de saude, estilos de vida e doenças crônicas—Brasil, Grandes Regiões e Unidades da Federação. 2014.

[44] WHO. WHO 2007 Growth reference data for 5–19 years. Geneva: World Health Organization; Available: http://www.who.int/growthref/en/. Accessed 26 October 2015.

[45] Fess E. Grip Strength, 2nd edition Chicago: American Society of Hand Therapists; 1992.

[46] Roberts HC, Denison HJ, Martin HJ, Patel HP, Syddall H, Cooper C, et al A review of the measurement of grip strength in clinical and epidemiological studies: towards a standardised approach. Age Ageing. 2011;40: 423–429. 10.1093/ageing/afr051 21624928

[47] Silva M, Badaró AFV, Dall’Agnol MM. Low back pain in adolescent and associated factors: A cross sectional study with schoolchildren. Brazilian J Phys Ther. 2014;18: 402–9. 10.1590/bjpt-rbf.2014.0051 PMC4228625 25372002

[48] Skoffer B. Low back pain in 15- to 16-year-old children in relation to school furniture and carrying of the school bag. Spine (Phila Pa 1976). 2007;32: E713–7. 18007232 10.1097/BRS.0b013e31815a5a44

[49] Oksuz E. Prevalence, risk factors, and preference-based health states of low back pain in a Turkish population. Spine (Phila Pa 1976). 2006;31: E968–72. 17139213 10.1097/01.brs.0000247787.25382.3c

[50] Shehab DK, Al-Jarallah KF. Nonspecific low-back pain in Kuwaiti children and adolescents: associated factors. J Adolesc Health. 2005;36: 32–5. 10.1016/j.jadohealth.2003.12.011 15661594

[51] Smith SM, Sumar B, Dixon KA. Musculoskeletal pain in overweight and obese children. Int J Obes. Nature Publishing Group; 2014;38: 11–15. 10.1038/ijo.2013.187 PMC3884137 24077005

[52] Shiri R, Karppinen J, Leino-Arjas P, Solovieva S, Viikari-Juntura E. The association between obesity and low back pain: A meta-analysis. Am J Epidemiol. 2010;171: 135–154. 10.1093/aje/kwp356 20007994

[53] Duryea S, Arends-Kuenning M. School Attendance, Child Labor and Local Labor Market Fluctuations in Urban Brazil. World Dev. 2003;31: 1165–1178. 10.1016/S0305-750X(03)00065-2

[54] Watson KD, Papageorgiou AC, Jones GT, Taylor S, Symmons DPM, Silman AJ, et al Low back pain in schoolchildren: the role of mechanical and psychosocial factors. Arch Dis Child. 2003;88: 12–17. 10.1136/adc.88.1.12 12495949 PMC1719294

[55] Rees CS, Smith AJ, O’Sullivan PB, Kendall GE, Straker LM. Back and neck pain are related to mental health problems in adolescence. BMC Public Health. BioMed Central Ltd; 2011;11: 382 10.1186/1471-2458-11-382 PMC3123209 21609488

[56] Standaert CJ, Herring SA, Pratt TW. Rehabilitation of the athlete with low back pain. Curr Sports Med Rep. 2004;3: 35–40. 14728912 10.1249/00149619-200402000-00007

[57] Cahalan R, O’Sullivan K, Purtill H, O’Sullivan P. a Cross-Sectional Study of the Biopsychosocial Characteristics of Elite Adult Irish Dancers and Their Association With Musculoskeletal Pain and Injury. Br J Sports Med. 2014;48: 576–576. 10.1136/bjsports-2014-093494.45

[58] El-Metwally A, Mikkelsson M, Ståhl M, Macfarlane GJ, Jones GT, Pulkkinen L, et al Genetic and environmental influences on non-specific low back pain in children: A twin study. Eur Spine J. 2008;17: 502–508. 10.1007/s00586-008-0605-1 18205017 PMC2295279

[59] O’Sullivan PB, Smith AJ, Beales DJ, Straker LM. Association of biopsychosocial factors with degree of slump in sitting posture and self-report of back pain in adolescents: a cross-sectional study. Phys Ther. 2011;91: 470–483. 10.2522/ptj.20100160 21350031

[60] O’Sullivan K, O’Keeffe M, O’Sullivan L, O’Sullivan P, Dankaerts W. Perceptions of sitting posture among members of the community, both with and without non-specific chronic low back pain. Man Ther. Elsevier Ltd; 2013;18: 551–556. 10.1016/j.math.2013.05.013 23806489

[61] Smith A, O’Sullivan P, Straker L. Classification of sagittal thoraco-lumbo-pelvic alignment of the adolescent spine in standing and its relationship to low back pain. Spine (Phila Pa 1976). 2008;33: 2101–2107. 18758367 10.1097/BRS.0b013e31817ec3b0

[62] Lis AM, Black KM, Korn H, Nordin M. Association between sitting and occupational LBP. European Spine Journal. 2007 pp. 283–298. 10.1007/s00586-006-0143-7 PMC2200681 16736200

[63] Dankaerts W, Sullivan PO, Burnett A, Straker L. Differences in Sitting Postures are Associated With Nonspecific Chronic Low Back Pain Disorders When Patients Are Subclassified. 2006;31: 698–704. 10.1097/01.brs.0000202532.76925.d2 16540876

[64] Wilke HJ, Neef P, Caimi M, Hoogland T, Claes LE. New in vivo measurements of pressures in the intervertebral disc in daily life. Spine (Phila Pa 1976). 1999;24: 755–762. 10222525 10.1097/00007632-199904150-00005

[65] O’Sullivan PB, Grahamslaw KM, Kendell M, Lapenskie SC, Möller NE, Richards K V. The effect of different standing and sitting postures on trunk muscle activity in a pain-free population. Spine (Phila Pa 1976). 2002;27: 1238–44. 12045525 10.1097/00007632-200206010-00019

[66] Shiri R, Karppinen J, Leino-arjas P, Solovieva S. The Association between Smoking and Low Back Pain : A Meta-analysis. AJM. Elsevier Inc.; 2010;123: 87.e7–87.e35. 10.1016/j.amjmed.2009.05.028 20102998

[67] Auvinen JP, Tammelin TH, Taimela SP, Zitting PJ. Is insufficient quantity and quality of sleep a risk factor for neck, shoulder and low back pain ? A longitudinal study among adolescents. 2010; 641–649. 10.1007/s00586-009-1215-2 PMC2899838 19936804

[68] Watson KD, Papageorgiou AC, Jones GT, Taylor S, Symmons DPM, Silman AJ, et al Low back pain in schoolchildren: Ocurrence and characteristics. Pain. 2002;97: 87–92. 12031782 10.1016/s0304-3959(02)00008-8

